# Effectiveness comparison of cardio-selective to non-selective β-blockers and their association with mortality and morbidity in end-stage renal disease: a retrospective cohort study

**DOI:** 10.1186/s12872-016-0233-3

**Published:** 2016-03-25

**Authors:** Theresa I. Shireman, Jonathan D. Mahnken, Milind A. Phadnis, Edward F. Ellerbeck

**Affiliations:** Health Services Policy & Practice and the Center for Gerontology & Health Care Research, Brown University School of Public Health, 121 South Main St, Box-G-S121-6, Providence, RI 02912 USA; Biostatistics, University of Kansas School of Medicine, Kansas City, KS USA; Preventive Medicine and Public Health, University of Kansas School of Medicine, Kansas City, KS USA; Medicine, University of Kansas School of Medicine, Kansas City, KS USA

**Keywords:** Dialysis, End stage renal disease, Hypertension, Mortality, β-blockers, Comparative effectiveness

## Abstract

**Background:**

Within-class comparative effectiveness studies of β-blockers have not been performed in the chronic dialysis setting. With widespread cardiac disease in these patients and potential mechanistic differences within the class, we examined whether mortality and morbidity outcomes varied between cardio-selective and non-selective β-blockers.

**Methods:**

Retrospective observational study of within class β-blocker exposure among a national cohort of new chronic dialysis patients (*N* = 52,922) with hypertension and dual eligibility (Medicare-Medicaid). New β-blocker users were classified according to their exclusive use of one of the subclasses. Outcomes were all-cause mortality (ACM) and cardiovascular morbidity and mortality (CVMM). The associations of cardio-selective and non-selective agents on outcomes were adjusted for baseline characteristics using Cox proportional hazards.

**Results:**

There were 4938 new β-blocker users included in the ACM model and 4537 in the CVMM model: 77 % on cardio-selective β-blockers. Exposure to cardio-selective and non-selective agents during the follow-up period was comparable, as measured by proportion of days covered (0.56 vs. 0.53 in the ACM model; 0.56 vs 0.54 in the CVMM model). Use of cardio-selective β-blockers was associated with lower risk for mortality (AHR = 0.84; 99 % CI = 0.72–0.97, *p* = 0.0026) and lower risk for CVMM events (AHR = 0.86; 99 % CI = 0.75–0.99, *p* = 0.0042).

**Conclusion:**

Among new β-blockers users on chronic dialysis, cardio-selective agents were associated with a statistically significant 16 % reduction in mortality and 14 % in cardiovascular morbidity and mortality relative to non-selective β-blocker users. A randomized clinical trial would be appropriate to more definitively answer whether cardio-selective β-blockers are superior to non-selective β-blockers in the setting of chronic dialysis.

## Background

Given their high rates of hypertension and cardiovascular disease (CVD) [1], patients with end-stage renal disease (ESRD) on chronic dialysis often are prescribed medications [2, 3] with cardioprotective properties [4–8]. In the general population, β-adrenergic blocking agents (hereafter referred to as β-blockers) are recommended as first and second line agents across a number of cardiac conditions because of their ability to reduce CVD events in at-risk individuals [9–11]. Risk reduction estimates from meta-analyses and systematic reviews of β-blockers range from a 16 % reduction in mortality in patients with diabetes to a 35 % reduction in mortality primarily from arrhythmia-associated sudden death [12–14].

The evidence base for the use of β-blockers in patients on chronic dialysis originates primarily from observational studies [15–21] and a few randomized trials [22–24], which generally reaffirm that these agents are associated with a therapeutic benefit. For example, using combined data from Dialysis Morbidity & Mortality Studies Waves 3 and 4, Foley and colleagues reported that β-blockers were associated with a 16 % relative risk reduction in all-cause mortality [15]. We also recently reported a significant reduction in all-cause mortality and cardiovascular endpoints associated with β-blockers in a propensity-adjusted modeling of time-dependent exposure [25, 26].

However, when making a choice to prescribe a specific cardioprotective medication for hypertension in the setting of chronic dialysis, providers have to make clinically-relevant selections from a given drug class without having the benefit of a clear evidence base. In the case of β-blockers, subclass distinctions in pharmacological properties, such as degree of β-1 or cardiac selectivity, α-blockade, route of elimination, lipophilicity, and dialyzability, become relevant; such a choice might be further influenced by presence of heart failure. For example, atenolol and metoprolol have high β-1 selectivity and low-to-moderate lipophilicity, making them readily dialyzable and placing them in distinction to carvedilol and labetalol. On the other hand, while theoretically carvedilol may offer advantages in hypertensive and/or heart failure patients [27, 28], observational studies have reported no significant differences in HF readmissions between agents in these subclasses of β-blockers [29, 30].

Within-class comparative effectiveness studies of β-blockers have not been performed in the chronic dialysis setting. In the absence of clinical trial data, well-designed observational studies can provide important preliminary data on the relative effectiveness of different medications, particularly in a patient population widely excluded from trials. The goal of the present study was to compare mortality and cardiovascular event outcomes across two major subclasses of β-blockers, focusing on cardiac selectivity distinctions between agents. To investigate this, we analyzed linked data from the United States Renal Data System (USRDS) with Medicaid pharmacy claims [2, 31] in a large cohort of incident dialysis patients who were newly initiating a β-blocker.

## Methods

### Study design and data sources

We performed a retrospective cohort analysis of incident, Medicare and Medicaid (dually eligible) chronic dialysis patients, quantifying their exposure to cardio-selective and non-selective β-blockers and assessing their outcomes over six years (2000–2005) [2, 31]. We used the dually eligible population because Medicare did not cover prescription medications during this time period. In addition, even with the implementation of drug coverage through Medicare Part D in 2006, medication exposure can still only be fully studied in the low-income subsidy patients (dually eligible), as many of these medications are filled through $4 prescriptions and claims are incomplete. Outcomes assessed were all-cause mortality and a combined outcome that included cardiovascular mortality and morbidity. The comparative effectiveness analyses were performed on new users of β-blockers as described below.

Data for these analyses were assembled from the USRDS and Medicaid (Centers for Medicare & Medicaid Services or CMS). From the USRDS, we obtained standard patient records that included information on demographics, comorbidities, functional status, and dialysis modality (from the Medical Evidence Form, or CMS 2728) recorded at the time of dialysis commencement. The USRDS also incorporated Medicare paid inpatient and outpatient medical claims, a federally-funded program for which the vast majority of adults with end stage renal disease are enrolled [1, 32]. We used Medicaid prescription drug claims to identify β-blocker exposure. Medicaid is a joint federal-state program designed to provide health care benefits to low-income persons: in the case of the dually eligible, Medicaid was the source of prescription drug coverage during the time period. These sources were linked using previously described methodology [31, 33] to permit identification of dually eligible dialysis patients in 2000–05.

### Cohort creation

We created a cohort consisting of hypertensive individuals who were new users of β-blockers. This included people who had at least one prescription for a β-blocker during the follow-up period, but no use during the first 90-day run-in period as is described below in greater detail. To assure complete observability of the cohort, we employed several criteria as have also been described elsewhere [31]. First, we limited the cohort to persons enrolled in a single state’s Medicaid fee-for-service program. Persons with coverage through the Veterans Administration and those who had previously been transplanted and returning to chronic dialysis were excluded. Persons who received a transplant, died, or were not continuously eligible for Medicare and Medicaid during the first 90 days on dialysis were excluded. Additionally, persons who did not fill any prescriptions during the first 90 days were excluded (this lack of prescriptions was thought to reflect the Medicaid’s spend-down requirements). Ohio residents were excluded since their claims do not include the days supplied of medication. We also excluded persons who were institutionalized during their entire follow-up period, were missing multiple data fields from their dialysis initiation Medical Evidence form (CMS 2728), and/or did not have hypertension documented on CMS 2728. Finally, we selected individuals who received at least one beta-blocker during their follow-up period.

The observation window began at the date the first β-blocker prescription was dispensed. Subjects were then followed until they incurred a first outcome event (death or cardiovascular event). They were censored when they lost Medicare or Medicaid eligibility, were transplanted, or reached the end of the observation window (12/31/2005).

### Covariates and descriptive variables

Demographic and clinical variables, drawn from the CMS 2728 form, included age, sex, race by ethnicity, employment status, smoking status (current at time of dialysis initiation), substance abuse (alcohol or illicit drugs), ability to ambulate and to transfer, body mass index (BMI), cause of ESRD, comorbidities, dialysis duration or vintage (before medication initiation), and dialysis modality. Ethnicity was categorized into one of four mutually exclusive groups: non-Hispanic Caucasians, non-Hispanic African-Americans, Hispanics, and Others. Body mass index (BMI) defined as dry weight was classified into 4 categories: < 20 kg/ m^2^, 20–24.99 kg/m^2^, 25–29.99 kg/m^2^, ≥ 30 kg/m^2^. Cause of ESRD was categorized as diabetes, hypertension, glomerulonephritis, or other. Comorbidities consisted of diabetes, congestive heart failure, coronary artery disease, cerebrovascular disease, and peripheral vascular disease. Because the CMS 2728 form is structured such that diabetes and hypertension may be considered either a cause of ESRD or a comorbidity, for the purposes of the present analysis, these two covariates were each considered a comorbidity if they were listed as either on the CMS 2728 form [34, 35]. Dialysis modality at time of dialysis initiation was categorized as in-center hemodialysis or self-care dialysis (home hemodialysis or peritoneal dialysis).

### Medication exposure

β-blockers were divided into two subclasses: cardio-selective (atenolol and metoprolol) and non-selective (carvedilol and labetalol). We excluded all other β-blockers as they accounted for fewer than 2 % of all prescriptions in a given year [33]. New β-blocker users were those who did not have any prescriptions for a β-blocker in the first 90 days following dialysis initiation. They also had to initiate use of β-blockers within the next 90-day window, e.g., days 91–180 on chronic dialysis, so as to limit bias from the potential accrual of new cardiovascular risks over time. Persons were assigned to a single β-blockers subclass: anyone who used medications from both subclasses of β-blockers during the follow-up period was excluded. However, switching was extremely rare: only 30 subjects switched from cardio-selective to non-selective agents and 28 subjects switched from non-selective to cardio-selective agent. Persons using other β-blockers were also excluded.

In order to determine whether the durations of exposure were comparable between β-blocker subclasses, we examined their proportion of days covered [36]. The proportion of days covered is computed from converting days supplied and dates from individual drug claims to a daily array. The proportion of days covered was adjusted for overlapping prescription fills, hospital, and skilled nursing facility days (since medications administered throughout the institutionalization would not result in an outpatient drug claim).

### Outcomes

All-cause mortality (ACM) was ascertained from the USRDS Core CD, which specifies the date and cause of death. In addition, we created a combined cardiovascular morbidity and mortality (CVMM) event outcome, capturing the first event per person. CVMM was defined as an inpatient hospitalization (Medicare Part A claims) for myocardial infarction (ICD-9 codes 410.x0, 410.x1), ischemic heart disease (411.xx), revascularization (ICD9 procedure codes 36.xx except 36.9), congestive heart failure (428.xx, 402.x1, 404.x1, or 404.x3), cerebrovascular accident (433.xx, 434.xx, 435.x), or peripheral vascular disease (440.2-4, 443.1, 443.81, 443.9, 444.2x, 444.81, 445.0x). Cardiovascular-related mortality was derived from the USRDS listed cause of death (myocardial infarction, atherosclerotic heart disease, cardiomyopathy, cardiac arrhythmia, cardiac arrest, cerebrovascular accidents). Outcome events were quantified as time from initiation of their β-blocker to either the event or censoring.

### Statistical analyses

To examine balance between subclasses (cardio-selective versus non-selective β-blockers) we generated contingency tables, comparing these groups using Pearson’s chi-square test and assessing validity by examining expected cell counts for categorical measures. For continuous measures, descriptive statistics were generated, stratified histograms were examined, and two-sample *t*-tests performed. To investigate within-class comparative effectiveness, we examined these data using Kaplan-Meier survival curves for an unadjusted comparison by stratifying by subclass. We then fit Cox proportional hazards regression models for ACM and CVMM outcome to compare the subgroups, adjusting for potential confounding through covariate adjustment. Model sample sizes were different for two reasons. A patient could have had a cardiovascular event before receiving a β-blocker, thus being eligible for the ACM model but not the CVMM model. Alternatively, a patient could have been on a both a cardio-selective and non-selective β-blocker during their time to mortality, but only a single subclass during their time to CVMM, thus being eligible for the CVMM model but not the ACM model.

Exponentiation of the parameter estimates obtained from these models using appropriate contrast statements allowed us to calculate the hazard ratios (HRs) for evaluating cardio-selective relative to non-selective β-blockers. Cox proportionality assumptions were ascertained through visual assessment of the complementary log-log survival plots.

Statistical significance was inferred when *P* < 0.01. All statistical analyses were done with SAS 9.2 (SAS Institute, Inc.).

### Sensitivity analyses

To test the robustness of our results, we performed several sensitivity analyses. First, we expanded the analysis to individuals who initiated cardio-selective versus non-selective β-blockers at any time while on dialysis. Second, to explore the potential impact of heart failure (HF) on our results, we modeled an interaction term between β-blocker subclass and HF as identified on the CMS 2728 form. This approach was selected because use of claims to determine true HF is particularly problematic in dialysis patients, with frequent misclassification of volume overload (typically resulting from inadequate ultrafiltration or missed dialysis treatments) as HF. Finally, we examined interaction terms for β-blocker subclass and race and coronary artery disease to verify the robustness of our analyses across these subpopulations.

### Ethics, consent and permissions

The research protocol received an expedited approval and HIPAA waiver by the institutional review board (Human Subjects Committee, #11436) at the University of Kansas Medical Center. Data Use Agreements (DUA) between the University and the USRDS (DUA #s: 2007–10, 2009–19, and 2015–2) and CMS (DUA #s: 16977 and 19707) permitted the data linking across the USRDS, Medicare and Medicaid files.

## Results

**Fig. 1:**
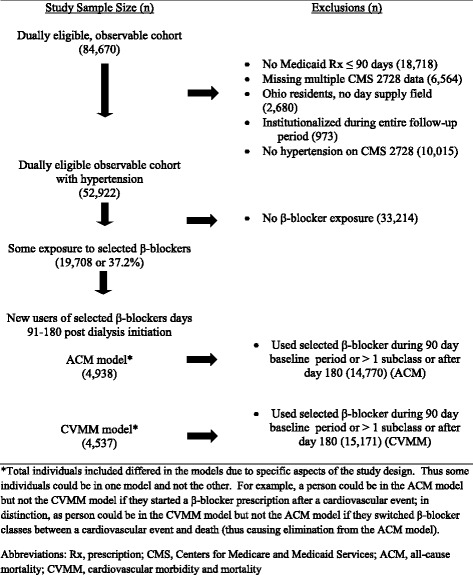
Flowchart demonstrating creation of the study cohort for new users of selected β-blockers

Of the initial 84,670 cohort, 52,922 with hypertension met criteria for observability (Fig. 1). More than one-third (37.2 % or 19,708) received a β-blocker prescription during their entire window of observation. There were 4938 who had no β-blocker use in the first 90 days on dialysis but started one in the next 90 days: they were included in the ACM model. The CVMM model sample was slightly smaller at 4537.

For the ACM model, new users included 3781 (76.6 %) who were exposed to atenolol or metopropolol (cardio-selective) and 1157 (23.4 %) who were exposed to carvedilol or labetalol (non-selective). In the CVMM model, 77.0 % of the new users were exposed to a cardio-selective β-blocker and the remaining 23.0 % received non-selective β-blockers. The baseline characteristics of the cohorts for both models are shown in Table 1. In both analytic cohorts (ACM and CVMM), non-selective β-blocker users were significantly younger by 2.1–2.5 years, less likely to be Caucasian and more likely to be African-American, and more likely to have heart failure. Primary cause of ESRD did not differ significantly between cardio-selective and non-selective β-blocker users in either model. The proportion of days covered for cardio-selective β-blockers was slightly higher (0.56 versus 0.54, *p* = 0.0043) in the ACM model, but they did not differ statistically in the CVMM Model. The overall distributions were quite comparable across both models, though, and as such we did not further adjust for proportion of days covered in the statistical models.

**Table 1:** Descriptive characteristics of new β-blockers medication users among chronic dialysis patients with hypertension across therapeutic subclasses

	All-cause mortality		CV event model	
	Cardio-selective	Non-selective	Cardio-selective	Non-selective
Number of cases	3781 (100 %)	1157 (100 %)	3495 (100 %)	1042 (100 %)
Age, mean years (SD)	60.4 (15.1)*	58.3 (15.9)*	60.1 (15.2)**	57.6 (16.0)**
Females, *n* (%)	2172 (57.5 %)	625 (54.0 %)	1992 (57.0 %)	559 (53.7 %)
Race/Ethnicity, *n* (%)				
African-American	1633 (43.2 %)*	580 (50.2 %)*	1531 (43.8 %)**	545 (52.3 %)**
Caucasian	1271 (33.6 %*	297 (25.7 %)*	1161 (33.2 %)**	246 (23.6 %)**
Hispanic	637 (16.9 %)*	212 (18.3 %)*	586 (16.8 %)**	191 (18.3 %)**
Other	240 (6.4 %)*	68 (5.9 %)*	217 (6.2 %)**	60 (5.7 %)**
BMI category, *n* (%)				
< 20 kg/m^2^	374 (9.9 %)	101 (8.7 %)	345 (9.9 %)	90 (8.6 %)
20–24.9 kg/m^2^	1101 (29.1 %)	355 (30.7 %)	1013 (29.0 %)	323 (31.0 %)
25–29.9 kg/m^2^	1004 (26.6 %)	320 (27.7 %)	936 (26.8 %)	279 (26.8 %)
30+ kg/m^2^	1255 (33.2 %)	369 (31.9 %)	1158 (33.1 %)	339 (32.5 %)
Missing	47 (1.2 %)	12 (1.0 %)	43 (1.2 %)	11 (1.0 %)
Current smoker, *n* (%)	284 (7.5 %)	63 (5.5 %)	261 (7.5 %)	55 (5.3 %)
Substance abuser,* *n* (%)	91 (2.4 %)*	47 (4.1 %)*	85 (2.4 %)**	47 (4.5 %)**
Unemployed, *n* (%)	3679 (97.3 %)	1129 (97.6 %)	3397 (97.2 %)	1014 (97.3 %)
Unable to ambulate, *n* (%)	207 (5.5 %)	52 (4.5 %)	196 (5.6 %)	47 (4.5 %)
Unable to transfer, *n* (%)	61 (1.6 %)	14 (1.2 %)	58 (1.7 %)	14 (1.3 %)
Cause of ESRD, *n* (%)				
Diabetes	2008 (53.1 %)	590 (51.0 %)	1837 (52.6 %)	530 (50.9 %)
Hypertension	1109 (29.3 %)	384 (33.2 %)	1031 (29.5 %)	350 (33.6 %)
Glomerulonephritis	304 (8.0 %)	84 (7.3 %)	287 (8.2 %)	75 (7.2 %)
Other	906 (9.9 %)	99 (8.6 %)	340 (9.7 %)	87 (8.4 %)
Comorbidities, n (%)				
Diabetes	2402 (63.5 %)	730 (63.1 %)	2198 (62.9 %)	646 (62.0 %)
Congestive heart failure	1287 (34.0 %)*	453 (39.2 %)*	1158 (33.1 %)**	399 (38.3 %)**
Coronary artery disease	1006 (26.6 %)	299 (25.8 %)	912 (26.1 %)	254 (24.4 %)
Peripheral vascular disease	585 (15.5 %)	151 (13.1 %)	536 (15.3 %)*	123 (11.8 %)*
Cerebrovascular accident	468 (12.4 %)	115 (9.9 %)	429 (12.3 %)	98 (9.4 %)
Modified Liu comorbidity	6.5 ± 3.6	6.61 ± 3.8	6.3 ± 3.6	6.4 ± 3.7
ACE/ARB use, baseline	37.3 %	35.4 %	36.7 %	34.4 %
CCB use, baseline	59.7 %*	61.9 %*	59.5 %**	63.2 %**
In-center hemodialysis *n* (%)	3582 (94.7 %)	1108 (95.8 %)	3313 (94.8 %)	995 (95.5 %)
Hemoglobin > = 11	893 (23.6 %)*	225 (19.5 %)*	822 (23.5 %)**	196 (18.8 %)**
Vintage (years) when drug initiated, mean (SD)	0.10 (0.07)	0.10 (0.07)	0.10 (0.07)	0.10 (0.07)
Proportion days covered, mean (SD)	0.56 (0.28)*	0.53 (0.28)*	0.56 (0.28)	0.54 (0.28)
Mortality, n (%)	1246 (33.0 %)	379 (32.7 %)		
CV event, n (%)			1627 (46.5 %)	485 (46.5 %)

*BMI* body mass index, *ESRD* end stage renal disease, *ACEI* angiotensin converting enzyme inhibitor, *ARB* angiotensin receptor blocker, *CCB* calcium channel blocker

*p < 0.01 for differences between subclasses within ACM model

**p < 0.01 for differences between subclasses within CVMM model

Nearly a third of each subclass cohort died, 33.0 % for cardio-selective β-blocker users and 32.7 % for non-selective β-blocker users. Cardiovascular causes accounted for 45.6 % of deaths: principally cardiac arrest with specific cause unknown (25.1 % of all deaths). CVMM rates (46.5 %) were also comparable for cardio-selective and non-selective β-blocker users. HF accounted for 53.3 % of hospitalization events, followed by CAD (including revascularization) at 20.8 %. Cerebrovascular events accounted for 14.6 % and peripheral vascular disease accounted for 11.3 % of hospitalizations.

**Fig. 2:**
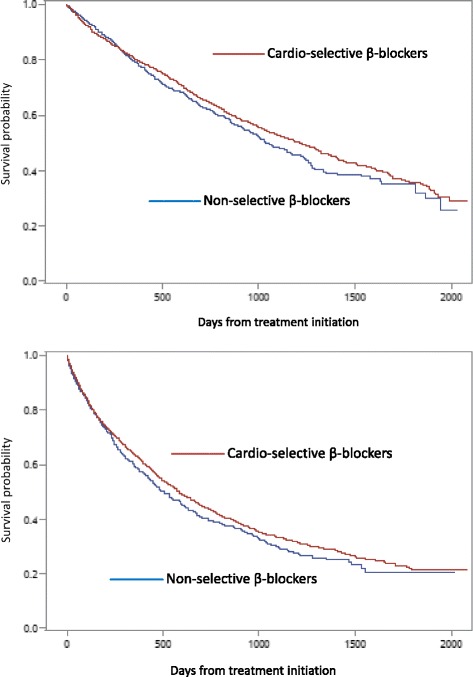
Kaplan-Meier survival curves for new users of beta-blockers. **a** All-cause mortality model (top) . **b** Cardiovascular morbidity and mortality model (bottom)

Survival time and time to CVMM events by the Kaplan-Meier method are shown graphically in Fig. 2a (ACM) & Fig. 2b (CVMM). For both ACM and CVMM, individuals prescribed cardio-selective β-blockers eventually had superior outcomes compared to those prescribed non-selective β-blockers. In the case of ACM, 50 % mortality was reached approximately 35.6 months for cardio-selective β-blockers users and 29.4 months for non-selective β-blocker users. For CVMM, 50 % CVMM events were reached approximately 19.7 months for cardio-selective β-blockers users and 15.8 months for non-selective β-blocker users.

Adjusted for all other factors (Table 2), use of cardio-selective β-blockers, compared to non-selective β-blocker use, was associated with a lower risk of mortality (AHR = 0.84; 99 % CI = 0.72–0.97, *p* = *0.0026*). Several other variables were significantly associated with a higher risk for mortality: age (AHR per decade = 1.15; 99 % CI = 1.00–1.33), Caucasian race (AHR = 1.29; 99 % CI = 1.10–1.33), low BMI (AHR = 1.38; 99 % CI = 1.11–1.72), comorbidity burden (AHR = 1.09; 99 % CI = 1.06–1.11), and self-care dialysis (AHR = 1.36; 99 % CI = 1.01–1.83). In the CVMM model, cardio-selective β-blockers were similarly associated with a lower risk for events (AHR = 0.86; 99 % CI = 0.75–0.99, *p* = *0.0042*). Only age (AHR per decade = 1.11; 99 % CI = 1.06–1.16) and the comorbidity burden (AHR = 1.07; 99 % CI = 1.04–1.09) were significantly associated with CVMM events.

**Table 2:** Comparative effectiveness of cardio-selective vs non-selective β-blockers in persons on chronic dialysis with respect to mortality (ACM) and cardiovascular morbidity-mortality (CVMM)

	ACM		CV event model	
	AHR	99 % CI	AHR	99 % CI
Cardio-selective vs. non-selective	0.84*	0.72–0.97	0.86*	0.75–0.99
Vintage (start of β-blocker)	1.71	0.69–4.28	0.85	0.38–1.90
Age, 10 year increments	1.15*	1.00–1.33	1.11*	1.06–1.16
Female sex	1.01	0.92–1.10	1.09	0.97–1.23
Race/Ethnicity				
Caucasian	1.29*	1.10–1.50	1.09	0.95–1.25
African-American	1.0	--	1.0	--
Hispanic	0.85	0.70–1.03	0.97	0.82–1.13
Other	0.79	0.59–1.06	0.98	0.77–1.25
BMI category				
< 20 kg/m^2^	1.38*	1.11–1.72	1.16	0.94–1.43
20–24.9 kg/m^2^	1.0	--	1.0	--
25–29.9 kg/.m^2^	0.84	0.71–1.00	1.01	0.87–1.17
30+ kg/m^2^	0.85	0.72–1.00	0.97	0.84–1.13
Missing BMI	1.00	0.55–1.80	1.18	0.81–1.73
Current smoker	1.05	0.83–1.38	1.18	0.71–1.96
Substance abuser	1.47	0.98–2.23	0.94	0.64–1.38
Unemployed	1.58	0.74–3.36	1.32	0.83–2.12
Inability to ambulate	1.25	0.93–1.69	1.00	0.75–1.32
Inability to transfer	1.20	0.72–1.99	0.85	0.50–1.46
Comorbidities				
Diabetes	0.93	0.80–1.09	1.04	0.91–1.19
Congestive heart failure	0.92	0.79–1.07	1.00	0.87–1.15
Coronary artery disease	1.04	0.89–1.21	1.06	0.93–1.22
Cerebrovascular accident	1.11	0.91–1.34	0.96	0.80–1.15
Peripheral vascular disease	1.00	0.83–1.20	1.09	0.92–1.29
Comorbidity burden (mod Liu)	1.09*	1.06–1.11	1.07*	1.04–1.09
Hemoglobin > = 11	1.00	0.86–1.17	0.96	0.84–1.11
Hemoglobin missing	1.05	0.83–1.33	0.98	0.80–1.20
Self-care dialysis	1.36*	1.01–1.83	1.16	0.90–1.50

*AHR* adjusted hazards ratio, *CI* confidence interval, *BMI* body mass index

*p < 0.01

In the sensitivity analyses using all new users β-blockers, the effectiveness of cardio-selective β-blockers was slightly higher (ACM model- AHR = 0.79; 99 % CI = 0.72–0.87, *P* < 0.0001: CVMM model AHR = 0.80; 99 % CI = 0.73–0.89, *P* < 0.0001). In separately run sensitivity analyses, the interaction term for HF and β-blocker subclass was not significant in either the ACM model (*p* = 0.72) or the CVMM model (*p* = 0.83). Tests for interactions between African-American race and β-blockers (ACM model, *p* = 0.90; CVMM model, *p* = 0.71) and coronary artery disease and β-blockers (ACM model, *p* = 0.30; CVMM model, *p* = 0.91) were also not significant. Accordingly, we reported the models, above, without the interaction terms.

## Discussion

With a theoretical potential for differential therapeutic effects, we examined within class effectiveness of cardio-selective versus non-selective β-blockers in patients on chronic dialysis. Among cohorts of new users of β-blockers, cardio-selective agents were associated with a significant 16 % reduction in mortality relative to non-selective β-blockers. A similar reduction in cardiovascular morbidity and mortality (14 %) was also noted in this large scale, observational study. Benefits were consistent regardless of underlying HF, CAD, and among African-American subjects.

Given the dearth of relevant literature, these findings constitute new insights for clinicians managing chronic dialysis patients. In an analysis of secondary data from Kaiser Permanente of Northern California, there were no significant differences in HF readmissions between metoprolol, atenolol, and carvedilol users with HF [29]. Only 5 % of the cohort was receiving chronic dialysis, and among those who were, most received either atenolol or metoprolol, similar to our cohort. A meta-analysis of carvedilol compared to β-1 selective agents netted eight trials, which when collectively analyzed, showed that carvedilol reduced all-cause mortality by 15 % in HF patients though there was not a significant reduction in HF readmissions [27]. Mortality benefits were higher (45 %) in AMI patients in three comparative trials but not consistently significant across fixed and random effect models. These studies, however, rarely included chronic dialysis patients, limiting their applicability to such patients.

It is widely appreciated that β-blockers are heterogeneous in their pharmacokinetics and potential mechanisms of action [37, 38]. In our analyses, we chose to focus on the clinical implications of the cardio-selectivity properties of β-blockers in the dialysis patient. Atenolol and metoprolol have greater β-1 selectivity and lower blood pressure through reducing cardiac output without effecting vascular resistance [27, 39]. In terms of pharmacokinetics, both are removed by dialysis. In contrast, carvedilol and labetalol effect α, β-1, and β-2 receptors and are not removed by dialysis [37, 38]. β-blockers appear to exert some of their impact centrally and impact vagal tone; this would appear to give theoretical advantages to the more lipophilic, non-selective agents [40]. Carvedilol, labetalol, and metoprolol are moderately lipophilic [37, 38]: the clinical implications of this would bias our findings toward the null since we considered metoprolol use in conjunction with atenolol. Furthermore, rapid changes in drug levels associated with dialysis of more hydrophilic agents such as atenolol might also be theorized to predispose patients to sympathetic overload during the peri-dialysis period [38]. These theoretical issues, however, were not confirmed in our study. In fact, we observed the opposite impact.

Carvedilol, in particular, has been singled out as the favored β-blocker in the dialysis setting [38]. The non-selective β-blockers offer α-blockade resulting in vasodilation and lower peripheral resistance without changes in cardiac output [27, 37, 38]. In addition, carvedilol reduces cardiac adrenergic activity while β-1 selective agents increase sensitivity to adrenergic activity [11]. Carvedilol may have pleiotropic effects (antioxidant & vasodilating) and antiarrhythmic effects which might lead to less sudden death [9]. β-2 receptors play a critical role in potassium influx into cells, and β-2 receptor antagonists can increase the risk of hyperkalemia [41]. The clinical implications of this have never been fully explored, even though patients on hemodialysis can have tremendous shifts in potassium both during and between dialysis episodes. Emerging data also suggest that β-2 stimulation may actually reduce apoptosis of damaged myocytes; a combination of β-1 blockade with certain β-2 agonists has demonstrated positive effects on ventricular remodeling in animal models [42]. This emerging data combined with the results of our observational data suggests the need for more extensive studies on the role of the β-2 receptor in patients with ESRD.

Our study has several important limitations. First, as an observational study, our investigation cannot prove causality. Only a randomized clinical trial would be able to definitively answer whether cardio-selective β-blockers are truly superior to non-selective β-blockers in reducing all-cause mortality and cardiovascular events. The most obvious treatment selection concern is the presence of heart failure and the theoretical advantages offered by carvedilol. While HF was slightly more prevalent in the non-selective cohort (about 5 % higher), there was no significant interaction between HF and β-blocker subclass. We did lack important patient-level clinical measures such as blood pressure level and ejection fractions. These factors might be unbalanced between the treatment groups and, therefore, be a source of residual confounding. Nevertheless, the majority of observed differences between treatment groups at baseline were minimal, and there was also no apparent therapeutic advantage in the first year or so of follow-up, which suggests that there were no major differences in baseline clinical factors. Any unmeasured, residual confounders would need to be both common and substantial to account for the large effect size that we observed in this study. We did contemplate a propensity adjustment, but the distributions of measured, baseline factors were so well-balanced that the approach would not have afforded much benefit.

We limited the look-back period for prior β-blocker use to 90 days to establish new use; this may be an imperfect approach, since patients may have been exposed to β-blockers in their more distant medical history, and therefore not been truly treatment naïve. We also limited the capture of new exposures to persons in the first six months of dialysis treatment so as to limit changes in underlying cardiovascular risks, but undoubtedly, subjects’ clinical status may have changed during this period of time. We did not include any measure of dose which might reflect the extent of β-blockade, but there is little reason to believe that with these new users, clinicians were using radically different dosing approaches across the two subclasses. While the study period is dated, 2000–2005, there have not been any major therapeutic breakthroughs within either subclass. Outpatient prescription medications were not covered under Medicare during this period, requiring us to use a Medicare-Medicaid eligible cohort. There is no physiologic reason to argue why dually enrolled beneficiaries would experience a different response as compared to the entire chronic dialysis population. In fact, our study cohort was younger and included more women and minorities than most Medicare only cohorts, providing greater generalizability. In addition, more contemporary studies of Medicare Part D prescription drug data would be limited as many β-blockers are available as $4 prescriptions which are not well captured in Part D claims. Other important strengths include use of a large sample size, employment of a design which focused on new users of the medications, demonstration of comparable levels of exposure between cardio-selective and non-selective β-blockers, and consistency in the results across sensitivity analyses.

## Conclusions

Among new initiating β-blocker users, chronic dialysis patients who received cardio-selective agents (metoprolol or atenolol) incurred a survival and cardiovascular endpoint advantage over their peers who received non-selective agents (carvedilol or labetalol). These findings may reflect the different mechanisms of action of these two medication subclasses. The initiation of dialysis is an appropriate time for providers to reconsider the ideal antihypertensive regimen for their patients. While it is unlikely that any pharmaceutical company would pursue a randomized clinical trial that compares subclasses of β-blockers in the dialysis population, the widespread use of these medications and their potential public health impact do point toward the need for a prospective comparative effectiveness trial.

## Abbreviations

ACM: all-cause mortality
AHR: adjusted hazard ratio
BMI: body mass index
CAD: coronary artery disease
CI: confidence interval
CMS: Centers for Medicare & Medicaid Services
CVD: cardiovascular disease
CVMM: cardiovascular morbidity and mortality
ESRD: end-stage renal disease
HF: heart failure
USRDS: United States Renal Data System
